# Acute phase response in bovine coronavirus positive post-weaned calves with diarrhea

**DOI:** 10.1186/s13028-019-0471-3

**Published:** 2019-07-25

**Authors:** Jeong-Byoung Chae, Jinho Park, Suk-Han Jung, Jin-Hee Kang, Joon-Seok Chae, Kyoung-Seong Choi

**Affiliations:** 1 Laboratory of Veterinary Internal Medicine, BK21 PLUS Program for Creative Veterinary Science Research, Research Institute for Veterinary Science and College of Veterinary Medicine, Seoul National University, Seoul, 08826 Republic of Korea; 2 Choong Ang Vaccine Laboratories Co., Ltd., CAVAC, Daejeon, 34055 Republic of Korea; 3 College of Veterinary Medicine, Chonbuk National University, Iksan, 54596 Republic of Korea; 4 National Institute of Animal Science, Rural Development Administration, Wanju, 53365 Republic of Korea; 5 College of Ecology and Environmental Science, Kyungpook National University, Sangju, 37224 Republic of Korea

**Keywords:** Bovine coronavirus, Haptoglobin, Serum amyloid A

## Abstract

Bovine coronavirus (BCoV) is associated with severe diarrhea in calves, winter dysentery in adult cattle, and respiratory diseases in cattle of all ages. This study aimed to investigate the relationship between white blood cell counts and haptoglobin (Hp) and serum amyloid A (SAA) levels in post-weaned calves with diarrhea caused by BCoV and those that recovered from diarrhea. Blood and fecal samples were collected twice from the same animals; 17 post-weaned calves with diarrhea (first) and 15 post-weaned calves that recovered from diarrhea (second). Real-time polymerase chain reaction revealed that all 17 fecal samples from post-weaned calves with diarrhea and one out of 15 from diarrhea-recovered calves were positive for BCoV and negative for *Cryptosporidium* spp., *Escherichia coli* K99, *Salmonella* spp., bovine rotavirus, and bovine viral diarrhea virus. No *Eimeria* oocysts were detected using the flotation method. In comparison with post-weaned calves with diarrhea, in diarrhea-recovered calves, the lymphocyte count was significantly higher (P = 0.018), and the monocyte count was significantly lower (P = 0.001); however, the number of monocytes was still high. Post-weaned calves with diarrhea had a significantly higher Hp concentration (P < 0.001) compared with diarrhea-recovered calves. The results indicated that increased Hp concentration and monocytosis but not SAA may be associated with diarrhea caused by BCoV. The present study suggests that the monitoring of Hp concentration and monocyte count is useful in the diagnosis of post-weaned calves with diarrhea caused by BCoV in this field.

## Findings

Bovine coronavirus (BCoV) is an enveloped, positive-sense single-stranded RNA virus belonging to the family *Coronaviridae*. BCoV is the causative agent of severe diarrhea in calves, winter dysentery in adult cattle, and respiratory disease in cattle of all ages [1, 2]. Infection leads to high morbidity, but usually low mortality rates. BCoV infection causes severe problems in cattle of various age groups, resulting in significant economic losses by affecting the weight gain or milk production. Clinical signs begin approximately 2 days after exposure and last for 3–6 days. Typically, BCoV infection in calves causes profuse watery diarrhea, and the feces can contain blood. BCoV replicates in the epithelial cells of colonic crypts, destroying the villi and leading to the degeneration, necrosis of the crypt epithelium, and petechial hemorrhage; this could result in severe, often hemorrhagic diarrhea in calves, which can be life-threatening due to the loss of electrolytes and malnutrition [2–4].

Acute phase proteins (APPs) are plasma proteins synthesized by e.g. hepatocytes in response to stress, infection, tissue injury or inflammation. APPs serve as the core of the innate immune response and are found in a variety of animal species [5]. Changes in the concentration of APPs have been recognized as a useful tool for assessment of cattle health. Haptoglobin (Hp) and serum amyloid A (SAA) are most common APPs in cattle [5, 6]. Several studies have reported changes in the concentration of APPs in several cattle diseases; however, few have focused on the relationship between calf diarrhea and APPs [7–9]. To date, there are no data available on the immunological changes in cattle naturally infected with BCoV. Therefore, the objective of this study was to investigate the relationship between white blood cell (WBC) counts and APP levels in the serum of post-weaned calves with clinical signs of diarrhea caused by BCoV and from the same calves after recovery from diarrhea.

All procedures were performed according to the ethical guidelines for the use of animal samples, as approved by the Chonbuk National University (Institutional Animal Care and Use Committee [IACUC] Decision No. CBU 2016-00026).

At a Holstein farm located at Jiri Mountain in the Republic of Korea, sudden onset of diarrhea was observed sporadically throughout the farm by the daily farm manager. This farm raised around 200 female Holstein post-weaned calves, which were consigned from the surrounding farms. In total, 17 post-weaned calves aged 117–155 days that had diarrhea for ≥ 3 days were randomly selected for the study. These weaned calves had no signs of respiratory disease.

Sampling was performed twice (October and December) from the same animals. First, 17 fecal samples were collected by rectal palpation on October 28th. Ten mL of blood (n = 17) was also taken from the jugular vein and equally divided into a K2 EDTA spray-dried anti-coagulated blood collection tube (BD Vacutainer^®^, Franklin Lakes, NJ, USA) and a SST blood tube (BD Vacutainer^®^). In the laboratory, the serum was separated by centrifugation at 3000*g* for 15 min. After 2 months (on December 22th), all post-weaned calves with previous BCoV associated diarrhea were diagnosed as healthy by a local veterinarian, and blood and fecal samples (n = 15) were taken. Samples could not be taken from two of the 17 calves, as they had already been moved to other farms. All fecal samples were stored at − 70 °C until analysis, and all serum samples were stored at − 20 °C until use. In total, 32 blood- and 32 fecal samples were collected.

Total DNA and RNA were extracted from fecal suspensions using MagMAX™ (Thermo Fisher Scientific, Waltham, MA, USA) according to the manufacturer’s instructions. Real-time polymerase chain reaction (PCR) was performed to detect *Cryptosporidium* spp., *Escherichia coli* K99, *Salmonella* spp., BCoV, bovine rotavirus (BoRVA), and bovine viral diarrhea virus (BVDV) using the Path-IDTM Multiplex One-Step RT-PCR Kit (Life Technologies, Carlsbad, CA, USA). All procedures were performed as previously described [10, 11].

Fecal samples were analyzed for the presence of oocysts using the flotation method with Sheather’s solution (saturated sugar solution; specific gravity = 1.28) and were examined microscopically (400× magnification) for *Eimeria* species.

Total WBC counts, including neutrophils, lymphocytes, monocytes, eosinophils, and basophils, were determined using an automatic blood analyzer (IDEXX ProCyte Dx, IDEXX Laboratories, Westbrook, ME, USA). The serum concentrations of Hp and SAA were measured using commercial enzyme-linked immunosorbent assay kits (Tridelta Development Ltd., Kildare, Ireland) according to the manufacturer’s instructions for cattle.

All statistical analyses (normality test and Wilcoxon signed-rank test) were performed using SPSS Statistics 25 for Windows (IBM, Armonk, NY, USA). All graphical procedures were performed using GraphPad Prism 6 (GraphPad, Software, San Diego, CA, USA).

All fecal samples (n = 17) collected at the end of October were from post-weaned calves with diarrhea. When the second set of samples (n = 15) was taken in December, none of the calves displayed diarrhea. All 17 fecal samples collected at the end of October and one fecal sample collected in December were positive for BCoV, while *Cryptosporidium* spp., *E*. *coli* K99, *Salmonella* spp., BoRVA, BVDV or *Eimeria* oocysts were not detected. The mean Ct value for BCoV in fecal samples from the 17 post-weaned calves with diarrhea was 22.7 (range 18.3–33.2), while the Ct value of the recovered calf was 34.2. These results indicated that not only post-weaned calves with diarrhea but also one recovered calf shed BCoV in the feces. BCoV is frequently associated with hemorrhagic diarrhea in calves [12, 13], however, none of the post-weaned calves in this study had hemorrhagic diarrhea. Our findings suggest that intestinal BCoV infection is not necessarily accompanied by hemorrhagic diarrhea in calves.

As shown in Table 1, in recovered post-weaned calves, the lymphocyte count was significantly increased (P = 0.018), and the monocyte count was significantly decreased compared with the count of calves with diarrhea (P = 0.001). The leukocyte, neutrophil, lymphocyte, and eosinophil counts were within reference ranges for post-weaned calves with diarrhea and those that recovered from diarrhea, whereas the monocyte count was above the reference range in both groups (Table 1). The delta measurements (values of post-weaned calves with diarrhea minus those of recovered calves) for lymphocyte and monocyte counts were significantly different in recovered calves. The total number of WBCs has been reported to decrease with age in cattle [14]. Previous studies have shown that from 4 to 6 months of age, the mean neutrophil count gradually declined, whereas the mean lymphocyte count had an increasing trend [15]. These findings are consistent with our results. There is a lack of data of the dynamics of different blood parameters for calves at this age range; however, in the present study, all WBCs except for monocytes were within the normal reference range [16]. The reason for the increase in monocytes is unclear in the present study. Nevertheless, a previous study showed that monocytosis could occur during exposure to acute stress and in the healing phase of acute and chronic infections [14]. However, the monocyte counts vary in cattle and thus are not a reliable indicator of a specific disease [17]. Further studies are needed to explore the association between monocytes and acute phase response (APR) and to evaluate the proinflammatory cytokines in animals infected with diarrhea.

**Table 1:** White blood cell parameters in post-weaned calves with diarrhea and in those that recovered from diarrhea

Parameters	Reference value	Diarrhea (*n* = 17)	Recovered (*n* = 15)	*P* value
WBC (10^3^/μL)	4.0–12.0	12.00 ± 0.86	10.17 ± 0.67	0.058
NEU (10^3^/μL)	0.6–4.1	3.31 ± 0.72	2.03 ± 0.22	0.148
LYM (10^3^/μL)	2.5–7.5	5.10 ± 0.30	6.28 ± 0.48	0.018*
MON (10^3^/μL)	0.0–1.2	3.49 ± 0.26	1.82 ± 0.12	0.001**
EOS (10^3^/μL)	0.0–2.4	1.00 ± 0.08	0.04 ± 0.01	0.248
BAS (10^3^/μL)	0.0–0.4	0.00 ± 0.00	0.00 ± 0.00	1

Parameters are the following: white blood cells (WBC), neutrophils (NEU), lymphocytes (LYM), monocytes (MON), eosinophils (EOS), basophils (BAS)

Normal reference values were established with data obtained from Schalm’s Veterinary Hematology [16]

Data are presented as the mean ± SEM. *P* values were obtained by Wilcoxon’s rank-sum test to compare post-weaned calves with diarrhea and diarrhea-recovered calves (* *P* < 0.05 and ** *P* < 0.01)

**Fig. 1:**
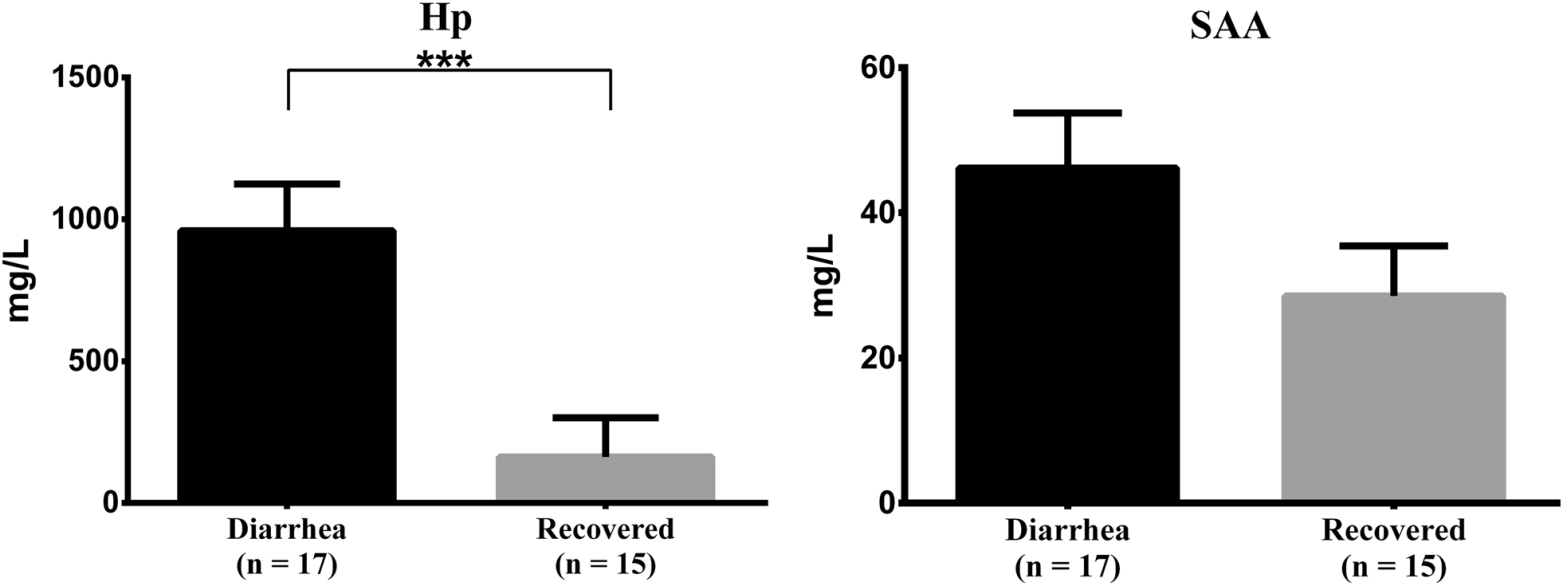
Haptoglobin (Hp) and serum amyloid A (SAA) concentrations (mean ± SEM) in the serum of post-weaned calves with diarrhea (n = 17) and calves that recovered from diarrhea (n = 15). A difference with a P value less than 0.05 according to Wilcoxon’s rank-sum test was considered significant (*P < 0.05, **P < 0.01, and ***P < 0.001)

The changes in the serum Hp and SAA concentrations are presented in Fig. 1. Hp concentration was higher in the post-weaned calves with diarrhea (932.0 ± 172.0 mg/L) than in the same calves recovered from diarrhea (162.2 ± 35.7 mg/L). Hp was 5.7-fold higher in post-weaned calves with diarrhea than in those that recovered from diarrhea. There was a significant difference in Hp concentration (P < 0.001) between post-weaned calves with diarrhea and recovered calves. Moreover, the concentration of SAA in post-weaned calves with diarrhea (46.2 ± 7.6 mg/L) was higher in recovered calves (28.5 ± 7.4 mg/L). SAA was increased 1.6-fold in post-weaned calves with diarrhea compared to the recovered calves; however, this increase was not statistically significant. The results showed that post-weaned calves with diarrhea had a significant increased Hp concentration. To the best of our knowledge, this is the first study to report the relationship between Hp concentration and BCoV infection. Our findings are consistent with those of previous studies, which reported that Hp is a major APP in cattle [6, 18]. SAA has been identified as an APP in various species [6]. However, compared with Hp, changes in SAA were less pronounced in post-weaned calves with diarrhea in this study. SAA has been reported to be more sensitive to stress than disease [19–21]. Although the number of samples used was limited and a definitive conclusion could not be provided, SAA may be less suitable for the assessment of cattle health, particularly for cattle with diarrhea caused by BCoV. Normally, the concentration of APPs increases when a disease develops and decreases during recovery [22]. However, in our study, SAA neither increased significantly in post-weaned calves with diarrhea nor did it decrease significantly during recovery. Taken together, the results suggested that serum Hp concentration may be a useful diagnostic indicator to differentiate between healthy and diseased animals. Further studies are needed to evaluate the association between other APPs (fibrinogen and α_1_ acid glycoprotein) and diarrhea caused by BCoV.

In conclusion, the present study is the first study that showed that post-weaned calves with diarrhea had monocytosis and a higher Hp concentration compared to recovered calves. Although we could not provide a definitive conclusion due to the limited number of samples, the increase in Hp concentration and monocyte count may be correlated with BCoV. The results suggest that the monitoring of the Hp response and monocytes in addition to clinical examination and PCR may be useful for prognostic assessment at least in post-weaned calves naturally affected with diarrhea caused by BCoV.

## Data Availability

The datasets used and/or analyzed during the current study are available from the corresponding author on reasonable request.

## Abbreviations

APPs: acute phase proteins
APR: acute phase response
BCoV: bovine coronavirus
BoRVA: bovine group A rotavirus
BVDV: bovine viral diarrhea virus
*E. coli*: *Escherichia coli*
EDTA: ethylenediaminetetraacetic acid
Hp: haptoglobin
SAA: serum amyloid A
real-time PCR: real-time polymerase chain reaction
WBC: white blood cell
